# Cross Species Association Examination of UCN3 and CRHR2 as Potential Pharmacological Targets for Antiobesity Drugs

**DOI:** 10.1371/journal.pone.0000080

**Published:** 2006-12-20

**Authors:** Zhihua Jiang, Jennifer J. Michal, Galen A. Williams, Tyler F. Daniels, Tanja Kunej

**Affiliations:** Department of Animal Sciences, Washington State University Pullman, Washington, United States of America; Institute for Genomic Research, United States of America

## Abstract

**Background:**

Obesity now constitutes a leading global public health problem. Studies have shown that insulin resistance affiliated with obesity is associated with intramyocellular lipid (IMCL) accumulation. Therefore, identification of genes associated with the phenotype would provide a clear target for pharmaceutical intervention and care for the condition. We hypothesized that urocortin 3 (*UCN3*) and corticotropin-releasing hormone receptor 2 (*CRHR2*) are associated with IMCL and subcutaneous fat depth (SFD), because the corticotropin-releasing hormone family of peptides are capable of strong anorectic and thermogenic effects.

**Methodology/Principal Findings:**

We annotated both bovine *UCN3* and *CRHR2* genes and identified 12 genetic mutations in the former gene and 5 genetic markers in the promoter region of the latter gene. Genotyping of these 17 markers on Wagyu×Limousin F_2_ progeny revealed significant associations between promoter polymorphisms and SFD (P = 0.0203−0.0685) and between missense mutations of exon 2 and IMCL (P = 0.0055−0.0369) in the bovine *UCN3* gene. The SFD associated promoter SNPs caused a gain/loss of 12 potential transcription regulatory binding sites, while the IMCL associated coding SNPs affected the secondary structure of *UCN3* mRNA. However, none of five polymorphisms in *CRHR2* gene clearly co-segregated with either trait in the population (P>0.6000).

**Conclusions/Significance:**

Because *UCN3* is located on human chromosome 10p15.1 where quantitative trait loci for obesity have been reported, our cross species study provides further evidence that it could be proposed as a potential target for developing antiobesity drugs. None of the markers in *CRHR2* was associated with obesity-type traits in cattle, which is consistent with findings in human. Therefore, CRHR2 does not lend itself to the development of antiobesity drugs.

## Introduction

Obesity has increased at a fast rate in recent years and is now a worldwide public health problem. The major consequence of overweight and obesity is that they are associated with more than 30 medical conditions, which cause approximately 300,000 deaths and total medical expenditures (direct and indirect) of $139 billion annually in the USA alone [1]. Insulin resistance, a characteristic of obesity, prevents insulin from taking the sugar from food and distributing it throughout the body for energy. Many studies have clearly indicated that intramyocellular accumulation of triglycerides is a major contributor to insulin resistance [2]. Therefore, identification of genes associated with intramyocellular lipid accumulation would provide a clear target for pharmaceutical intervention and care for obesity and its related conditions, such as high blood pressure, type 2 diabetes, coronary heart disease, some types of cancer, poor female reproductive health and psychological disorders.

Urocortin 3 (UCN3) and corticotropin-releasing hormone receptor 2 (CRHR2) are members of the corticotropin-releasing hormone (CRH) family of peptides. UCN3 binds selectively to CRHR2 [3] and both are co-expressed throughout the central nervous system, such as in the ventromedial hypothalamic nucleus, lateral septum and bed nucleus of the stria terminalis [4], as well as in the gastrointestinal tract [5]. Both UCN3 and CRHR2 are, therefore, thought to play a central role in appetite and gastrointestinal motor regulation. Indeed, when exposed to a high fat diet, *CRHR2*-mutant mice consumed significantly more food while maintaining the same body weight as their wild-type littermates [6]. Intracerebroventricular injections of UCN3 were found to reduce appetite by suppressing food intake in the freely-fed rat [7].

On the other hand, there is increasing evidence supporting the involvement of these two peptides in the regulation of energy homeostasis and in mediating the anorexic effect of CRH at the adipose level. For example, Seres and colleagues [8] found that both *UCN3* and *CRHR2* are expressed in human visceral and subcutaneous adipose tissue. Obviously, the local production of these two peptides within the adipose tissue indicates their direct involvements in fat cell function in addition to their central effects on weight regulation. In particular, Doyon and colleagues [9] concluded that CRHR2 could be a potential target for the development of an antiobesity drug. Thus, we hypothesized that genetic polymorphisms of *UCN3* and *CRHR2* genes are associated with intramyocellular lipid accumulation (IMCL) and subcutaneous fat depth (SFD) in mammals. In order to test the hypothesis, we annotated bovine *UCN3* and *CRHR2* genes and identified a total of 17 genetic polymorphisms for an association study. Statistical analysis using the general linear model (GLM) procedure of SAS and quantitative transmission-disequilibrium test (QTDT) revealed that *UCN3* gene, but not its receptor *CRHR2* gene, is significantly associated with both intramyocellular and subcutaneous lipid accumulation in Wagyu×Limousin F_2_ crosses.

## Materials and Methods

### Animals and phenotypic traits

A Wagyu×Limousin reference population was developed, including 6 F_1_ bulls, 113 F_1_ dams and ∼250 F_2_ progeny [10]. The Japanese Wagyu breed of cattle has been traditionally selected for high IMCL accumulation (measured as marbling score with an average of 8.52), whereas the Limousin breed has been selected for heavy muscle, which leads to low IMCL accumulation (average marbling score less than 4.78) [11]. The difference in IMCL accumulation between these two breeds makes them very unique for mapping QTLs for the trait. Beef marbling is the term commonly used to describe the appearance of white flecks or streaks of fat between the muscle fibers in meat, which is essentially equivalent to IMCL accumulation measured in humans. Beef marbling score was a subjective measure of the amount of IMCL in the *longissimus* muscle based on USDA standards (http://www.ams.usda.gov/). Subcutaneous fat depth (SFD) was measured at the 12–13^th^ rib interface perpendicular to the outside surface at a point three-fourths the length of the longissimus muscle from its chine bone end. The marbling scores for IMCL ranged from 4 to 9.5 and SFD varied from 0.1 to 1.3 inches in the population.

### Sequence annotation and primer design

We determined the genomic organization of bovine *UCN3* and *CRHR2* by aligning a bovine cDNA sequence (BC114855) with a bovine genomic DNA contig (AAFC03043460) for the former gene, and aligning the human mRNA sequence (NM_001883) with a bovine genomic DNA contig (AAFC03056271) for the latter gene. Three pairs of primers were designed to target the promoter (forward–5′GGG GCT GCA CCA AGC AAA TGT CAA C3′ and reverse–5′TCT ACC CTT CTT CCT GGA GCC AAC3′), non-coding exon 1 (forward – 5′AGG TCT GGG AGA GAA GGT GGG TAG3′ and reverse – 5′AAA CAC AGA CAT TGA CGG TTC AGC3′) and coding exon 2 (forward – 5′CTG AAC TTG CAC AAA GCC TGG TAG3′ and reverse – 5′CCC AGC CTC CTC CTC TAC TTC TTC3′) in the *UCN3* gene. An additional two pairs of primers were designed to amplify products in the promoter (forward – 5′TGA GAC TGG AGC ACA CAA ACA CAG3′ and reverse – 5′CAA GTG TGG AGG AGC TGA AAA CCT3′) and exon 1 region (forward – 5′TCC TCT CCG CTA AGG TCC AGA CT3′ and reverse – 5′ AGG AAC ACT CAC GGG TCG TGT TAT3′) in the bovine *CRHR2* gene, respectively.

### Polymorphism detection and genotyping assay development

Approximately 50 ng of genomic DNA each from six F_1_ bulls were amplified in a final volume of 10 µL that contained 12.5 ng of each primer, 150 µM dNTPs, 1.5 mM MgCl_2_, 50 mM KCl, 20 mM Tris-HCl and 0.25 U of Platinum Taq polymerase (Invitrogen, Carlsbad, CA). The PCR conditions were carried out as follows: 94°C for 2 min, 32 cycles of 94°C for 30 sec, 63°C for 30 sec and 72°C for 30 sec, followed by a further 5 min extension at 72°C. PCR products were then sequenced for polymorphism detection on an ABI 3730 sequencer in the Laboratory for Biotechnology and Bioanalysis (Washington State University) using a standard protocol. The same PCR product direct sequencing approach was also used to genotype the polymorphisms on all animals.

### Data analysis

The estimates for degrees of Hardy-Weinberg equilibrium within each mutation and linkage disequilibrium between mutations and selection of tagging genetic polymorphisms in each of bovine *UCN3* and *CRHR2* genes were performed using the HAPLOVIEW program [12]. The phenotypic data for both IMCL and SFD measurements were previously adjusted for year of birth, sex, age (days), live weight (kilograms), or fat depth (inches), as appropriate. The adjusted phenotypes were then used in a subsequent association analysis using the GLM (general linear model) procedure of SAS v9.1 (SAS institute Inc., Cary, NC). Pair-wise comparisons of least squares means were performed using a protected t-test. Additionally, quantitative transmission disequilibrium test (QTDT) [13] was performed to further examine the association between the tagging mutations and adjusted obesity-related phenotype data. *P* value <0.05 was considered statistically significant after Bonferroni correction. For significantly associated mutations, the MatInspector web server [14] was used to screen potential transcriptional regulatory binding site changes caused by promoter polymorphisms, while the Mfold web server [15] was used to predict mRNA secondary structure changes caused by coding polymorphisms.

## Results

### Genomic organization of the bovine *UCN3* and *CRHR2* genes

**Figure 1:**
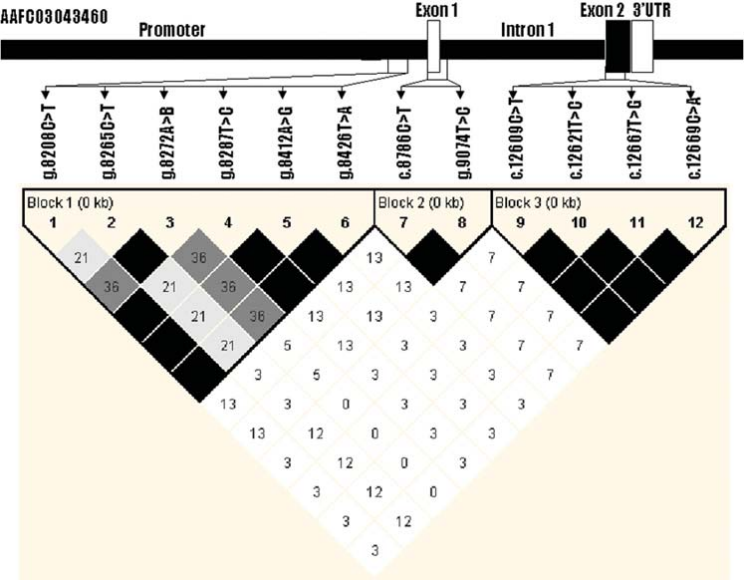
Genomic organization and haplotype analysis in the bovine *UCN3* gene. Noncoding exon 1, partial non-conding exon 2 and 3′untranslated region are marked by white boxes and coding exon 2 by a black box. Pairwise linkage disequilibrium relationship for 12 mutations is illustrated based on r^2^ measurements. The mutation g.8272A>B represents AAFC03043460.1:*g.8272-8281AATAATAAAT>GGAGC*.

BLAST searches using the cDNA sequence of the human *UCN3* gene (NM_053049) as a reference retrieved three bovine orthologous cDNA sequences from the GenBank database. The longest cDNA sequence BC114855 with 1,404 bp was used and retrieved one genomic DNA sequence (AAFC03043460) of the same gene from the 7.15× bovine genome sequence database. Alignment of both cDNA and genomic DNA sequences determined the genomic organization of the bovine *UCN3* gene. Like all four human *CRH* paralogs, the bovine *UCN3* gene has two exons and one intron (Figure 1).

**Figure 2:**
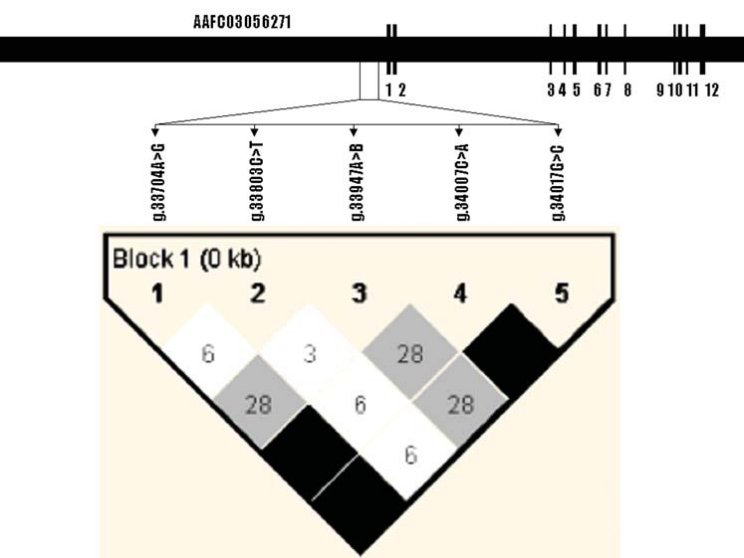
Genomic organization and haplotype analysis in the bovine *CRHR2* gene. Coding exons are marked by black boxes. Pairwise linkage disequilibrium relationship for 5 mutations is illustrated based on r^2^ measurements. The mutation g.2072A>B represents AAFC03056271.1:*g.33947-33964TGAATCCAGCCTGAGTTG>CTTTGTCTTGAG*.

For the bovine *CRHR2* gene, a BLAST search using the human mRNA sequence (NM_001883) retrieved three bovine orthologous ESTs (BI849955, DV873120 and CK774717), but they could not form a full-length cDNA sequence for the bovine gene. Fortunately, one bovine genomic DNA contig (AAFC03056271) from the 7.15× bovine genome sequence database was obtained using the human cDNA sequence, and alignment of the human cDNA sequence and the bovine genomic DNA sequence unraveled the genomic organization of the bovine *CRHR2* gene, including the promoter region. The genomic organization of *CRHR2* gene is also conserved in cattle, which consists of 12 exons and 11 introns (Figure 2).

### Single and multiple nucleotide polymorphisms

In the bovine *UCN3* gene, the promoter region harbors one multiple nucleotide polymorphism (MNP) and five single nucleotide polymorphisms (SNPs), while exons 1 and 2 contain two and four SNPs, respectively (Figure 1). The MNP has two homozygous alleles of 10 bp and 5 bp, i.e., AAFC03043460.1:*g.8272-8281AATAATAAAT>GGAGC*. The remaining eleven SNPs are AAFC03043460.1:*g.8208C>T, g.8265C>T, g.8287T>C, g.8412A>G, g.8426T>A, c.8786C>T, g.9074T>C, c.12609C>T, c.12621T>C, c.12667T>G* and *c.12669C>A*, respectively (Figure 1). Among these five coding SNPs, two (*c.12667T>G* and *c.12669C>A*) are missense mutations and both occur in one codon (codon 59), changing phenylalanine (*TTC*) to valine (*GTA*) at the preprohormone level of the UCN3 peptide. One MNP and four SNPs were detected in the promoter region of bovine *CRHR2* gene (Figure 2). The MNP possesses two homozygous alleles of AAFC03056271.1:*g.33947-33964TGAATCCAGCCTGAGTTG>CTTTGTCTTGAG* with 18 bp in one allele and 12 bp in other allele. Four SNPs include AAFC03056271.1:*g.33704A>G, g.33803C>T, g.34007C>A and g.34017G>C*, respectively. No polymorphism was detected in the exon 1 region of bovine *CRHR2* gene.

### Haplotype analysis and selection of tagging mutations

In the bovine *UCN3* gene, sequencing of 6 F_1_ sires indicated that four SNPs in the promoter region: *g.8208C>T, g.8287T>C, g.8412A>G* and *g.8426T>A* form two haplotypes: *CTAT* and *TCGA*. Two SNPs in the exon 1 and flanking regions *c.8784C>T* and *g.9072T>C* also appear in two haplotypes: *CC* and *TT* in the population, while all four SNPs (*c.12609C>T, c.12621T>C, c.12667T>G* and *c.12669C>A*) in coding exon 2 region have no historical recombination in either *CTTC* or *TCGA* haplotypes. The lack of historical recombination among SNPs in each of these regions described above was further confirmed by the HAPLOVIEW program on genotype data of all F_2_ progeny (Figure 1). Therefore, *g.8208C>T, g.8265C>T*, *g.8272-8281AATAATAAAT>GGAGC, c.8784C>T*, and *c.12669C>A* were chosen as tagging mutations for association analysis. Among five mutations in the promoter region of bovine *CRHR2* gene, HAPLOVIEW indicated AAFC03056271.1:*g.33704A>G, g.34007C>A and g.34017G>C* have no-historical recombination by forming two haplotypes of *GAC* and *ACG* (Figure 2), and thus three mutations including AAFC03056271.1:*g.33704A>G, g.33803C>T*, and *g.33947-33964TGAATCCAGCCTGAGTTG>CTTTGTCTTGAG* were selected as tagging mutations for association analysis.

### Association analysis of *UCN3* and *CRHR2* genes with IMCL and SFD

Two statistical approaches–the general linear model (GLM) and the quantitative transmission disequilibrium test (QTDT) were used to detect associations between genetic polymorphisms in both bovine *UCN3* and *CRHR2* genes with IMCL and SFD in a reference population of Wagyu×Limousin F_2_ cross cattle (Table 1). Overall, the reference population had an average SFD of 0.394 inches with a standard deviation of 0.18 inches. In the bovine *UCN3* gene, GLM analysis indicated a suggestive association between genotype at *g.8208C>T* and SFD (P = 0.0685), while the QTDT test indicated a significant association between the genotype and SFD (P = 0.0203; Table 1). Animals with *TT* genotypes had 0.086 (P = 0.0045) and 0.056 inches (P = 0.0259) less subcutaneous fat than animals with *CC* and *CT* genotypes, which account for 0.48 and 0.31 standard deviations for the trait, respectively (Table 1).

**Table 1:**
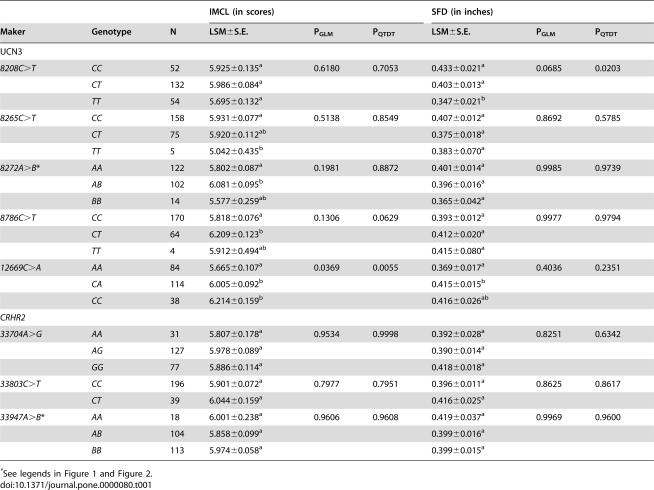
Associations of *UCN3* and *CRHR2* genes with IMCL and SFD

			IMCL (in scores)			SFD (in inches)		
Maker	Genotype	N	LSM±S.E.	P_GLM_	P_QTDT_	LSM±S.E.	P_GLM_	P_QTDT_
UCN3								
*8208C>T*	*CC*	52	5.925±0.135^a^	0.6180	0.7053	0.433±0.021^a^	0.0685	0.0203
	*CT*	132	5.986±0.084^a^			0.403±0.013^a^		
	*TT*	54	5.695±0.132^a^			0.347±0.021^b^		
*8265C>T*	*CC*	158	5.931±0.077^a^	0.5138	0.8549	0.407±0.012^a^	0.8692	0.5785
	*CT*	75	5.920±0.112^ab^			0.375±0.018^a^		
	*TT*	5	5.042±0.435^b^			0.383±0.070^a^		
*8272A>B* *	*AA*	122	5.802±0.087^a^	0.1981	0.8872	0.401±0.014^a^	0.9985	0.9739
	*AB*	102	6.081±0.095^b^			0.396±0.016^a^		
	*BB*	14	5.577±0.259^ab^			0.365±0.042^a^		
*8786C>T*	*CC*	170	5.818±0.076^a^	0.1306	0.0629	0.393±0.012^a^	0.9977	0.9794
	*CT*	64	6.209±0.123^b^			0.412±0.020^a^		
	*TT*	4	5.912±0.494^ab^			0.415±0.080^a^		
*12669C>A*	*AA*	84	5.665±0.107^a^	0.0369	0.0055	0.369±0.017^a^	0.4036	0.2351
	*CA*	114	6.005±0.092^b^			0.415±0.015^b^		
	*CC*	38	6.214±0.159^b^			0.416±0.026^ab^		
*CRHR2*								
*33704A>G*	*AA*	31	5.807±0.178^a^	0.9534	0.9998	0.392±0.028^a^	0.8251	0.6342
	*AG*	127	5.978±0.089^a^			0.390±0.014^a^		
	*GG*	77	5.886±0.114^a^			0.418±0.018^a^		
*33803C>T*	*CC*	196	5.901±0.072^a^	0.7977	0.7951	0.396±0.011^a^	0.8625	0.8617
	*CT*	39	6.044±0.159^a^			0.416±0.025^a^		
*33947A>B* *	*AA*	18	6.001±0.238^a^	0.9606	0.9608	0.419±0.037^a^	0.9969	0.9600
	*AB*	104	5.858±0.099^a^			0.399±0.016^a^		
	*BB*	113	5.974±0.058^a^			0.399±0.015^a^		

* See legends in Figure 1 and Figure 2.

Overall, IMCL accumulation, described by marbling scores, for all F_2_ progeny averaged 5.916 with a standard deviation of 1 marbling score. Interestingly, the genotype effects on IMCL accumulation increased in significance with mutations closer to the coding regions of the *UCN3* gene (Table 1). The *12669C>A* marker was significantly associated with IMCL (P = 0.0369 for the GLM analysis and P = 0.0055 for the QTDT test, respectively). *AA* animals were much leaner, with 0.549 and 0.340 lower marbling scores, respectively than *CC* animals (P = 0.0045) and *CT* heterozygotes (P = 0.0164) (Table 1). Unfortunately, none of the markers in the bovine *CRHR2* gene were associated with either IMCL or (P>0.60) (Table 1).

### Functional characterization of promoter and coding polymorphisms associated with SFD and IMCL

**Figure 3:**
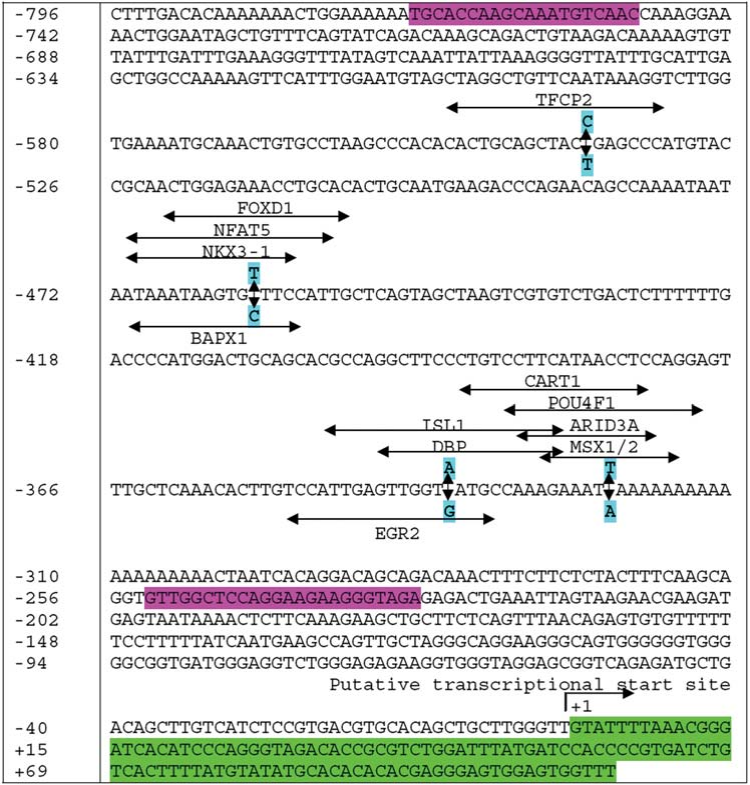
Nucleotide sequence of the proximal promoter region of the bovine *UCN3* gene. Primer and partial non-coding exon 1 sequences are shadowed by pink and bright green color, respectively. The putative transcription start site is numbered as +1. Four polymorphic sites that were associated with SFD are bold and shadowed by turquoise color. Potential transcription regulatory binding sites for TFCP2, NFAT5, NKX3-1, FOXD1, ISL1, DBP, CART1, POU4F1, ARID3A and MSX1/MSX2 are associated with haplotype *CTAT*, while only binding sites for BAPX1 and EGR2 are linked to haplotype *TCGA*.

As indicated above, only mutations in the bovine *UCN3* gene were significantly associated with either trait in the reference population. Therefore, it might be interesting to characterize how the promoter polymorphisms affect transcriptional regulatory binding sites and how coding polymorphisms have an impact on the mRNA secondary structure. In the promoter region of the bovine *UCN3* gene, four polymorphisms, AAFC03043460.1:*g.8208C>T, g.8287T>C, g.8412A>G* and *g.8426T>A* form two haplotypes: *CTAT* and *TCGA*. MatInspector [14] detected a remarkable difference in the number of potential transcriptional regulatory binding sites between these haplotypes: ten for the former haplotype, while only two for the latter haplotype (Figure 3). These twelve transcriptional binding sites were for TFCP2 (transcription factor CP2), NFAT5 (nuclear factor of activated T-cells 5, tonicity-responsive), NKX3-1 (NK3 transcription factor related, locus 1), FOXD1 (forkhead box D1), BAPX1 (bagpipe homeobox homolog 1), ISL1 (ISL1 transcription factor, islet-1), DBP (D site of albumin promoter binding protein), EGR2 (early growth response 2), CART1 (cartilage paired-class homeoprotein 1), POU4F1 (POU domain, class 4, transcription factor 1), ARID3A (AT rich interactive domain 3A) and MSX1 (msh homeobox homolog 1)/MSX2 (msh homeobox homolog 2), respectively (Figure 3).

**Figure 4:**
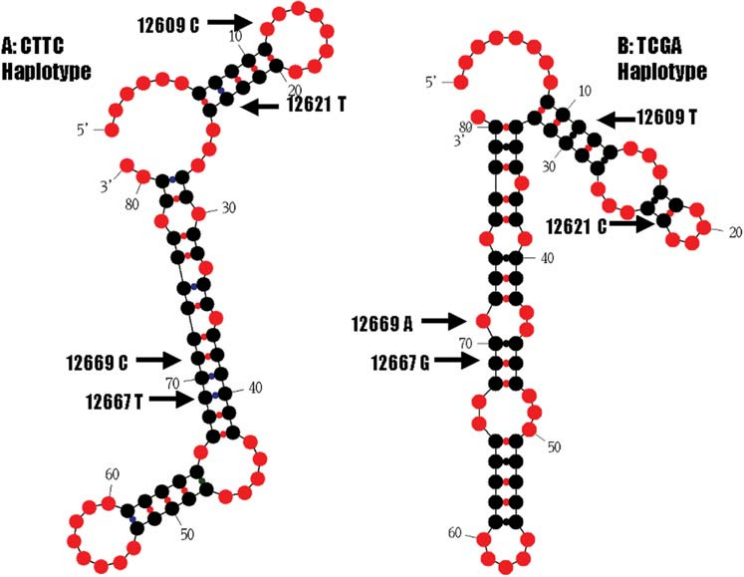
*UCN3* mRNA secondary structure predicted by Mfold on a partial sequence of 81 bp surrounding four coding SNPs. A: mRNA secondary structure for haplotype *CTTC*. B: mRNA secondary structure for haplotype *TCGA*.

Four coding SNPs in exon 2 of bovine *UCN3* gene: AAFC03043460.1:*c.12609C>T, c.12621T>C, c.12667T>G* and *c.12669C>A* also form two haplotypes: *CTTC* or *TCGA*. We used the Mfold program [15] to predict how these two haplotypes affect mRNA secondary structure. In the first run, a complete coding sequence of 501 bp for the preprohormone was used in the analysis. The sequences with both haplotypes were folded with Mfold in a locally automated manner. The complete coding sequence containing *CTTC* haplotype yielded a total of 13 secondary structures, while the sequence with the *TCGA* haplotype produced a total of 16 secondary structures. However, there was a difference in single-strandedness counts (ss-counts) between two haplotypes. The ss-counts measure the number of times each nucleotide is unpaired across all predicted secondary structures. For the former haplotype, 122 of 501 bp had zero ss-counts; while for the latter haplotype, 100 of 501 had zero ss-counts across all predicted secondary structures (Fisher's exact test, P = 0.0150). In the second run, we selected 81 bp of sequence surrounding the SNPs for a more localized structure analysis. As showed in Figure 4, both haplotypes had a strong effect on the secondary structure of bovine *UCN3* mRNA.

## Discussion

There are four paralogous corticotropin-releasing hormone genes in mammalian genomes: corticotropin-releasing hormone, urocortin, urocortin 2 and urocortin 3 [16]. In the present study, we revealed several interesting features about urocortin 3 in the bovine genome. First, the bovine *UCN3* gene region seems highly polymorphic. We designed three pairs of primers that amplified a total of 1,679 bp. A total of 12 mutations (one approximately every 140 bp of sequence) were detected in this region. Second, a multiple nucleotide polymorphism was detected in the promoter region of the bovine *UCN3* gene. One allele has 10 nucleotides of *AATAATAAAT*, while another has only five nucleotides of *GGAGC*. No significant similarity could be determined between these two alleles. Third, two SNPs (*c.12667T>G* and *c.12669C>A*) occurred in one codon (codon 59), leading to a change from phenylalanine (*TTC*) to valine (*GTA*) at the preprohormone level of UCN3 peptide. Both SNPs only form two haplotypes–*TC* and *GA*, showing no historical recombination in the population. Lastly, HAPLOVIEW analysis revealed that three amplified regions hold three haplotype blocks (Figure 1); although the amplified promoter region and exon 1 region are just 147 bp apart and exon 1 and exon 2 regions are just 3,209 bp apart. These data might provide a foundation for further investigation on formation and evolution of *CRH* paralogs in mammals.

More importantly, we found that the *UCN3* gene is significantly associated with IMCL accumulation and SFD (Table 1). However, four promoter SNPs organized into two haplotyes had a strong association with SFD, while four SNPs that also formed two haplotypes in exon 2 yielded a strong association with IMCL accumulation. In the SFD analysis, animals with *TT* genotypes of *g.8208C>T* had 0.086 (P = 0.0045) and 0.056 inches (P = 0.0259) less subcutaneous fat than animals with *CC* and *CT* genotypes, which account for 0.48 and 0.31 standard deviations for the trait. In the IMCL analysis, *AA* animals at position *c.12669C>A* had 0.549 and 0.340 lower marbling scores than *CC* animals (P = 0.0045) and *CT* heterozygotes (P = 0.0164). On the other hand, the *AA* animals tended to be leaner with 0.047 and 0.046 less inches of SFD compared to the *CC* and *CA* genotypes (Table 1), which approached the significance level (P = 0.0982 for the GLM analysis and P = 0.0522 for the QTDT test when the P values were uncorrected). These data indicate that increasing SFD with a promoter polymorphism does not necessarily result in an increase of IMCL accumulation. However, it is very likely that increasing IMCL with the exon 2 polymorphisms would also stimulate high accumulation of SFD, and thus lead to an overall increase of whole body fat deposition. As intramyocellular lipid accumulation in muscle is a major contributor to both insulin resistance and whole body fat deposition, inhibiting IMCL gain should be a long-term goal for preventing obesity in human.

In the human genome, *UCN3* is placed at position 5.40 Mb on 10p15.1, where two independent studies suggested quantitative trait loci (QTL) for body mass index (BMI) in Pima Indians [17] and in Caucasians [18]. Interestingly, both groups used the same flanking markers–*D10S1435* and *D10S189*, spanning from 2.23 Mb to 6.76 Mb on human chromosome 10. Obviously, the *UCN3* gene should be a strong candidate gene for the human BMI QTL detected in the region, as our current study provided strong evidence supporting its involvement in regulation of lipogenesis. In the study, we developed five genetic markers in the promoter region of the bovine *CRHR2* gene, but none were significantly associated with either IMCL accumulation or SFD in Wagyu×Limousin F_2_ cross cattle (Table 1).

This was not surprising because no association of *CRHR2* gene has been observed with obesity in humans. Challis and associates [19] screened 51 severely obese children (body mass index (BMI)>4 kg/m^2^ standard deviations above the age-related mean), a UK Caucasian population-based cohort for genetic polymorphisms in the human *CRHR2* gene. In subjects with extreme early-onset obesity, three missense mutations were found in *CRHR2* (Glu220Asp, Val240Ile and Val411Met). However, none of these missense mutations clearly cosegregated with obesity in family studies. A common single-nucleotide polymorphism *G1047A* (Ser349Ser) was also detected in *CRHR2*, but it was not associated with any obesity-related phenotype. The authors concluded that mutations in the coding sequence of the *CRHR2* gene are unlikely to be a common monogenic cause of early-onset obesity. Therefore, the association studies conducted in cattle and in human failed to provide any evidence supporting *CRHR2* as a potential target for the development of an antiobesity drug, as proposed by Doyon and colleagues [9].

The remarkably different expression patterns between *UCN3* and *CRHR2* genes in adipocyte tissue and skeletal muscle might provide some hints on why the former, not the latter gene, is associated with SFD and IMCL accumulation observed in the present study. In the human subcutaneous fat tissue, quantitative expression analysis revealed that *UCN3* mRNA is expressed approximately four fold higher than its receptor, *CRHR2* mRNA [8]. *UCN3* mRNA is expressed in the skeletal muscle of adult mammals and *Xenopus laevis*, but no one has detected any expression of *CRHR2* mRNA in the tissue of any species [20]. Therefore, the lower expression or no expression of *CRHR2* mRNA in these tissues might lead to its limited effects on fat cell function and muscle thermogenesis. On the other hand, evidence has shown that UCN3 is directly involved in regulation of glucagons and insulin secretion [21]. Injection of murine synthetic UcnIII into male rats significantly increased both blood and insulin levels. UCN3 also stimulated glucagons and insulin release from the isolated rat islets. In the present study, the high SFD associated haplotype *CTAT* in the promoter region gained 10 new transcriptional regulatory binding sites in comparison with the other haplotype of *TCAT* in the bovine *UCN3* gene. Among these 10 transcriptional regulatory binding sites, three are for TFCP2 (transcription factor CP2), NKX3-1 (NK3 transcription factor related, locus 1) and NFAT5 (nuclear factor of activated T-cells 5, tonicity-responsive). Studies have shown that these three genes may affect the risk of Alzheimer's disease [22], prostate cancer [23] and diabetic nephropathy [24], conditions often associated with obesity. All these data clearly support that the *UCN3* gene plays an important role in regulation of adipocyte metabolism through a broad pathway.

In conclusion, we annotated the bovine *UCN3* and *CRHR2* genes using a comparative approach and developed a total of 17 genetic markers in both genes. Genotyping these markers on ∼250 Wagyu×Limousin F_2_ crosses revealed that the bovine *UCN3*, but not its receptor *CRHR2* gene, is significantly associated with intramyocellular lipid accumulation and subcutaneous fat depth in cattle. The promoter polymorphisms of the bovine *UCN3* gene alter 12 potential transcription regulatory binding sites, some of which are associated with obesity-related conditions. The coding polymorphisms of the gene affect the secondary structure of *UCN3* mRNA remarkably. Therefore, we propose UCN3 as a strong target for developing antiobesity drugs. However, the candidacy of CRHR2 for the purpose needs to be further evaluated.
